# Association of Cognitive Function With Event‐Related Potentials and Immune Inflammation in Adolescents With Major Depressive Disorder

**DOI:** 10.1002/brb3.71802

**Published:** 2026-10-18

**Authors:** Lu Yang, Liemin Ruan, Zhongze Lou, Zhenglai Liu, Yiqin Huang, Li Zhang, Yunxin Ji

**Affiliations:** ^1^ Department of Psychosomatic Medicine The First Affiliated Hospital of Ningbo University Ningbo Zhejiang China

**Keywords:** adolescents, cognitive impairment, event‐related potentials, inflammatory cytokines, major depressive disorder, T lymphocyte subsets

## Abstract

**Objective:**

This study aimed to investigate cognitive function changes in adolescents with major depressive disorder (MDD) and examine their associations with depression severity, event‐related potentials (ERP), T lymphocyte subsets, and inflammatory cytokines to identify exploratory clinical and biological correlates for cognitive impairment in this population.

**Methods:**

We enrolled 89 adolescent patients with MDD and 30 healthy controls, matched for age and gender. Cognitive function was assessed using the Montreal Cognitive Assessment Scale (MoCA), depression severity with the Hamilton Depression Scale (HAMD17), and anxiety with the Hamilton Anxiety Scale. ERP were recorded, and blood samples were analyzed for T lymphocyte subsets and inflammatory cytokines.

**Results:**

Adolescents with MDD exhibited significantly lower MoCA scores, indicating cognitive impairment, particularly in attention, delayed recall, and executive function. ERP component analysis showed prolonged latencies and decreased amplitudes in the MDD group. Increased serum concentrations of interleukin‐1 beta and interleukin‐6, and elevated percentages of CD3+CD4+ and CD3+CD8+ cells were observed in the depressive group, with positive correlations between CD3+ cells and MoCA scores and between CD3+CD8+ cells and HAMD17 scores.

**Conclusion:**

Adolescents with MDD showed lower cognitive performance, which was associated with depression severity and immune‐inflammatory indicators.

## Graphical abstract

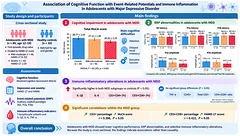

## 1 Introduction

Depressive disorder is a common and debilitating psychiatric condition characterized by persistent low mood, anhedonia, and reduced cognitive function. In adolescents, these symptoms not only hinder academic performance and social development but are also associated with functional impairment and cognitive difficulties that may persit across illness stages (Varghese et al. 2022; Schilling et al. 2021). Recent epidemiological studies show that depression is emerging at younger ages, with a prevalence of 0.8% to 1.6% among school‐age children (Thapar et al. 2022). A recent global systematic review and meta‐analysis further reported that approximately one in five children and adolescents experience depression or depressive symptoms, highlighting the growing public health burden of adolescent depression (Lu et al. 2024).

The causes of adolescent depression are multifactorial, involving genetic vulnerability (Li et al. 2022), environmental adversity (Toenders et al. 2022), and neurobiological changes associated with puberty (Stumper and Alloy 2023). However, a growing body of evidence highlights the role of neuroinflammation in the onset and persistence of depressive symptoms. Pro‐inflammatory cytokines such as interleukin‐1 beta (IL‐1β), interleukin‐6 (IL‐6), and tumor necrosis factor‐alpha (TNF‐α) have been associated with alterations in neurotransmitter systems, neuroplasticity, and brain connectivity, processes relevant to mood regulation and cognition (Toenders et al. 2022; Sun et al. 2022). Importantly, depressive symptoms, cognitive impairment, and immune‐inflammatory abnormalities should not be regarded as independent pathological phenomena. Rather, prior studies suggest that these domains may be interrelated, potentially through overlapping neurobiological pathways. Increased depressive severity has been reported alongside inflammatory pathway activation, and inflammatory markers have been associated with hippocampal and prefrontal cortical functions relevant to attention, memory, and executive function. Conversely, cognitive dysfunction may be associated with poorer emotional regulation and daily functioning, which could coexist with greater depressive symptom burden and immune‐inflammatory alterations.

Several studies have demonstrated the link between inflammation and depression. For instance, a study by Zhang et al. (2023) found that adolescent patients with first‐episode, drug‐naive major depressive disorder (MDD) exhibited significantly higher levels of IL‐1β and IL‐6 compared to healthy controls. These elevated cytokine levels were associated with cognitive deficits, particularly in attention and executive function. Similarly, Chen et al. (2024) reported that pro‐inflammatory cytokines were elevated in adolescents and young adults with MDD, and these levels correlated with the severity of depressive symptoms and suicidal ideation. These findings suggest that immune‐inflammatory activation may be associated with both depressive symptom burden and cognitive impairment in adolescents. From this perspective, more severe depression may be associated with stronger inflammatory responses, and these immune changes may be linked to delayed information processing, reduced attentional allocation, and poorer cognitive performance. Therefore, examining depression severity, immune‐inflammatory markers, and cognitive function within the same analytical framework may help characterize the complex neuroimmune associations observed in adolescent depression.

Despite these insights, few studies have integrated electrophysiological and immune‐inflammatory markers to characterize cognitive dysfunction in adolescents with depression. The relationship between these biological factors and cognitive symptoms, such as attention and executive deficits, is still poorly defined. Therefore, this study aims to fill this gap by investigating the association between cognitive function, event‐related potentials (ERPs), and immune‐inflammatory indicators in adolescents with MDD. ERPs are non‐invasive neurophysiological tools that provide millisecond‐level temporal resolution to assess cognitive processing (Hyder et al. 2020). Specifically, the N2 component is associated with conflict monitoring and attentional control (Perez‐Osorio et al. 2021), P3a reflects automatic orienting to novel stimuli, and P3b is linked to conscious allocation of attentional resources and working memory updating (Kruiper et al. 2019). These ERP components are widely used as candidate objective indicators of cognitive function and are especially relevant for populations with attention and executive function deficits, such as adolescents with depression. Therefore, we selected N2, P3a, and P3b to capture multiple dimensions of cognitive dysfunction in this group. On this basis, the present study was designed to address the following research question: whether cognitive impairment in adolescents with depressive disorder is associated not only with depression severity but also with electrophysiological abnormalities and immune‐inflammatory dysregulation. We hypothesize that cognitive impairment in this population is associated with abnormal ERP responses and dysregulated inflammatory activity. By exploring these associations, we hope to provide preliminary evidence on neuroimmune correlates of cognitive decline and identify candidate biomarkers for early intervention research in adolescent depression.

## 2 Method

### 2.1 Study Population

This study enrolled 89 adolescent patients with MDD and 30 age‐ and gender‐matched healthy controls from the First Affiliated Hospital of Ningbo University between January 2023 and June 2024. The age range of the MDD group was 12–18 years. The MDD group included patients who met the following criteria: (1) age between 12 and 18 years; (2) diagnosis of MDD, single episode, according to the Diagnostic and Statistical Manual of Mental Disorders, Fifth Edition (DSM‐5); (3) Hamilton Depression Scale (HAMD17) scores ≥8; and (4) first onset of MDD and no previous antidepressant, antipsychotic, mood stabilizer, or systematic psychotherapy treatment before enrollment.

The diagnosis was made independently by two experienced psychiatrists based on DSM‐5 diagnostic criteria and clinical interviews. To avoid diagnostic ambiguity in early‐onset depressive symptoms, special attention was paid to the differential diagnosis between MDD and bipolar disorder. Patients were excluded if they had any current or lifetime history of manic, hypomanic, or mixed episodes; a previous diagnosis of bipolar disorder or related disorders; psychotic symptoms; or clinical features strongly suggestive of bipolar spectrum disorder. Information regarding manic or hypomanic symptoms, psychiatric history, treatment history, and family history of bipolar disorder was obtained from both patients and their guardians. When the two psychiatrists disagreed on the diagnosis, the case was discussed until consensus was reached.

Exclusion criteria for the MDD group included bipolar disorder and related disorders, schizophrenia spectrum disorders or other psychotic disorders, confirmed cardiovascular and cerebrovascular diseases, including coronary heart disease, cerebrovascular disease, peripheral vascular disease, hypertension, diabetes, hyperlipidemia, drug or alcohol dependence, trauma, ulcers, retinopathy, surgery within the last 3 months, and inflammatory or tumor diseases. Patients with a history of neurological disorders, intellectual disability, severe suicidal risk requiring emergency intervention, or any medication use that could affect cognitive function or immune parameters within the last month were also excluded. Patients who were pregnant, breastfeeding, or menstruating at the time of blood sampling were also excluded.

#### 2.1.1 Inclusion and Exclusion Criteria for the Control Group

The healthy control group consisted of 30 adolescents recruited from local schools and community centers in the Ningbo area, matched for age and gender with the patient group. Inclusion criteria for the control group were as follows:

1. Age between 12 and 18 years.
2. No history of psychiatric disorders, as confirmed by a clinical interview.
3. HAMD17 score <8 and Hamilton Anxiety Scale (HAMA) score <7, indicating the absence of significant depressive or anxiety symptoms.
4. No history of chronic medical conditions such as cardiovascular disease, diabetes, or neurological disorders.
5. No use of medications known to affect cognitive function or immune parameters in the past month.

Exclusion criteria for the control group included:

1. History of any psychiatric disorder or significant psychological distress.
2. Presence of any chronic medical condition that could influence cognitive function or immune status.
3. Use of any medication known to affect cognitive function or immune parameters in the past month.
4. History of substance abuse or dependence.
5. Pregnancy, breastfeeding, or menstruation (for females).

Both groups were matched for socio‐economic status (SES), based on parental education level and family income, as well as urban/rural residence to minimize potential confounding effects on cognitive function. All participants and their guardians provided informed consent, and the study was approved by the Ethics Committee of the First Affiliated Hospital of Ningbo University (Ethics Approval No. 2022 Research No. 047A).

As this was an exploratory observational study in adolescents with MDD, no a priori sample size calculation was conducted due to limited existing data on ERP and immune biomarkers in this population. However, a post hoc power analysis based on the primary outcome (Montreal Cognitive Assessment Scale [MoCA] score difference between groups) indicated that the current sample size achieved a statistical power of over 0.80 with *α* = 0.05 and medium effect size (Cohen's *d* = 0.5), suggesting that the sample was sufficient to detect meaningful differences. Future studies will incorporate formal sample size planning based on pilot data.

### 2.2 Group Classification

Participants with MDD were further classified into two subgroups based on their HAMD17 scores: (1) Mild‐to‐moderate MDD group: HAMD17 scores ranging from 8 to 23; (2) Severe MDD group: HAMD17 scores ≥24. The control group consisted of healthy individuals with HAMD17 scores <8 and HAMA scores <7, confirming the absence of significant depressive or anxiety symptoms. Thus, the diagnostic label used in this study was MDD rather than a broad, unspecified depressive disorder label.

### 2.3 Psychological and Neurophysiological Assessment

Psychiatrists collected data on patients' medical history, age, gender, height, body mass index, and blood pressure. Psychological scales were assessed by two trained psychiatrists. The HAMD17 assessed depression severity, the HAMA assessed anxiety, and the Montreal Cognitive Assessment—Beijing version (MoCA) evaluated cognitive function (Sun et al. 2022). The MoCA is a widely used brief cognitive screening instrument, and recent validation work supports its utility for detecting global cognitive impairment (McDicken et al. 2019). The Kappa value of the consistency test of the above scales evaluated by two doctors was 0.90.

ERP were recorded using the NeuroScan SynAmps2 EEG system (Compumedics, USA). Electrodes were placed according to the 10–20 international system. The Oddball auditory paradigm was employed: 80% standard tones (1000 Hz) and 20% target tones (2000 Hz), intensity 80 dB, delivered binaurally via headphones. Participants responded to target tones via button press. EEG signals were filtered (0.1–30 Hz), and artifacts exceeding ±100 µV were excluded. Trials with over 20% response errors were discarded.

Latency and amplitude values of N2, P3a, and P3b components were extracted and averaged over 250 valid trials. The ERP data were analyzed using NeuroScan software (Curry 8.0).

### 2.4 Blood Sample Collection and Detection

Fasting blood samples were collected between 7:00 and 8:00 a.m. using standard vacuum tubes. Serum was separated by centrifugation (3000 rpm, 15 min, 4°C) and stored at −80°C.

T lymphocyte subsets (CD3+, CD4+, CD8+, and CD45+) were assessed via flow cytometry (BD FACSCanto II, BD Biosciences, USA) and analyzed using FlowJo v10 software.

Inflammatory cytokines IL‐1β, IL‐6, and TNF‐α were measured using ELISA kits (Thermo Fisher Scientific; Cat. No. KHC0011, KHC0061, KHC0031) per the manufacturer's protocol.

### 2.5 Statistical Methods

SPSS 25.0 software package was used for statistical analysis of all data. Continuous variables were tested for normality using the Kolmogorov–Smirnov test and for homogeneity of variance using Levene's test. All variables met the assumptions for parametric testing (*p* > 0.05). Measurement data were presented as mean ± standard deviation (±s), and comparisons of group means were performed using one‐way analysis of variance (ANOVA). Categorical variables were presented as frequencies or percentages and analyzed using the chi‐square (χ^2^) test. Spearman correlation analysis was employed to examine associations between cognitive function, depression/anxiety severity, and inflammatory markers. A significance level of *P* < 0.05 was used, and Bonferroni correction was applied when conducting multiple comparisons to control for type I error. Because this study used a cross‐sectional observational design, the statistical analyses were primarily intended to identify associations rather than establish causal relationships. Therefore, the observed correlations among depression severity, immune‐inflammatory markers, ERP parameters, and cognitive function should be interpreted as exploratory associations. Given the limited sample size and the absence of longitudinal measurements, formal causal mediation or moderation analysis was not performed in the present study. Future studies with larger samples and repeated follow‐up assessments are needed to test whether immune‐inflammatory abnormalities mediate or moderate the relationship between depression severity and cognitive impairment.

## 3 Result

### 3.1 Comparison of Demographic and Baseline Characteristics

There was no statistical significance in the comparison of general data between the two groups (*P* > 0.05), as shown in Table 1.There were no significant differences between the two groups in terms of age, gender distribution, body mass index (BMI), years of schooling, or place of residence (urban vs. rural) (*P* > 0.05). This indicates that the groups were well matched at baseline, allowing for a more accurate comparison of the study outcomes.

**TABLE 1:** Comparison of general data between patient group and control group.

	Mild‐to‐moderate depression group (61 cases)	Major depression group (28 cases)	Healthy control group (30 cases)	*F*/x^2^	*p* value
Age (years)	14.66 ± 1.68	14.89 ± 1.37	14.10 ± 1.81	1.839	0.164
Gender (female/male)	46/15	22/6	23/7	0.107	0.948
Body mass index (kg/m^2^)	16.63 ±2.56	16.11 ± 1.90	15.84 ± 2.91	1.108	0.334
One‐child ratio	34/61	19/28	20/30	1.668	0.434
Years of schooling (years)	7.56 ± 1.34	7.61 ± 1.07	6.97 ± 1.10	2.787	0.066
Nation	All Han				
Occupation	All students				
Place of residence (urban/rural)	45/16	19/9	23/7	0.600	0.741
Religious belief	None				
Marital status	Unmarried				

Data are presented as mean ± standard deviation or counts. *p*‐values were calculated using ANOVA for continuous variables and chi‐square test for categorical variables. No significant differences were found between groups (*p* > 0.05).

### 3.2 Comparison of Cognitive Function, Depression Severity, and Anxiety Severity Among the Three Groups

Table 2 presents the results of the cognitive function assessment (MoCA), depression severity (HAMD17), and anxiety severity (HAMA) for the three groups: mild‐to‐moderate MDD, severe MDD, and healthy controls. The results show significant differences among the groups: the severe MDD group had the highest mean HAMD17 score (27.96 ± 3.82), followed by the mild‐to‐moderate MDD group (17.82 ± 3.89), with the healthy control group having the lowest score (3.37 ± 1.35). The differences among the groups were statistically significant (*F* = 383.557, *p* < 0.001). Similar to HAMD17, the severe MDD group had the highest mean HAMA score (27.18 ± 7.25), followed by the mild‐to‐moderate MDD group (18.57 ± 5.82), and the healthy control group had the lowest score (3.47 ± 1.28). The differences among the groups were statistically significant (*F* = 141.764, *p* < 0.001). The healthy control group had the highest mean MoCA score (28.77 ± 1.14), indicating better cognitive function. The mild‐to‐moderate MDD group had a mean MoCA score of 27.11 ± 1.39, while the severe MDD group had the lowest score (24.25 ± 2.19). The differences among the groups were statistically significant (*F* = 62.295, *p* < 0.001). The progressive decline in MoCA scores from healthy controls to mild/moderate and then to severe depression is consistent with an association between cognitive impairment and depression severity.

**TABLE 2:** Comparison of HAMD17, HAMA, and MoCA scores among the three groups.

	Mild‐to‐moderate depression group	Major depression group	Healthy control group	*F* value	*p* value
HAMD17	17.82 ± 3.89	27.96 ± 3.82	3.37 ± 1.35	383.557	0.000**
HAMA	18.57 ± 5.82	27.18 ± 7.25	3.47 ± 1.28	141.764	0.000**
MoCA	27.11 ± 1.39	24.25 ± 2.19	28.77 ± 1.14	62.295	0.000**

^*^Data are presented as mean ± standard deviation. *p*‐values were calculated using ANOVA. *p < 0.001 indicates significant differences between groups. Abbreviations: HAMA, Hamilton Anxiety Scale; HAMD17, 17‐item Hamilton Depression Scale; MoCA, Montreal Cognitive Assessment Scale.

### 3.3 Analysis of ERP Components

Table 3 presents the detailed results of the ERP components analysis, comparing the mild‐to‐moderate MDD group, the severe MDD group, and the healthy control group. The results show significant differences in ERP components between the groups: The mean latency of N2 was significantly prolonged in the mild‐to‐moderate depression group compared to the healthy controls (217.19 ± 22.40 vs. 193.45 ± 14.11 ms; *F* = 12.107, *p* < 0.001). Further analysis revealed that the mild‐to‐moderate depression group had a longer N2 latency compared to the severe MDD group (217.19 ± 22.40 vs. 212.28 ± 26.74 ms; *p* < 0.05). The mean latency of P3a was prolonged in the MDD group compared to the healthy controls (293.74 ± 25.55 vs. 277.91 ± 9.35 ms; *F* = 6.353, *p* = 0.002). The severe depression group showed a longer P3a latency compared to the mild‐to‐moderate MDD group (295.67 ± 23.02 vs. 293.74 ± 25.55 ms; *p* < 0.05). The mean latency of P3b was prolonged in the MDD group compared to the healthy controls (348.35 ± 42.28 vs. 324.76 ± 6.55 ms; *F* = 6.841, *p* = 0.002). The severe depression group had a longer P3b latency compared to the mild‐to‐moderate MDD group (357.04 ± 35.63 vs. 348.35 ± 42.28 ms; *p* < 0.05). The mean amplitude of P3a was significantly decreased in the MDD group compared to the healthy controls (6.08 ± 4.62 vs. 8.80 ± 1.12 µV; *F* = 3.320, *p* = 0.040). The severe depression group had a lower P3a amplitude compared to the mild‐to‐moderate MDD group (4.67 ± 3.41 vs. 6.08 ± 4.62 µV; *p* < 0.05). The mean amplitude of P3b was decreased in the MDD group compared to the healthy controls (4.67 ± 3.41 vs. 7.39 ± 1.31 µV; *F* = 4.995, *p* = 0.008). The severe depression group showed a lower P3b amplitude compared to the mild‐to‐moderate MDD group (4.67 ± 3.41 vs. 6.74 ± 4.15 µV; *p* < 0.05). Overall, the MDD groups exhibited prolonged ERP latencies and reduced amplitudes, suggesting slowed cognitive processing and reduced neural engagement; however, the direction of the N2 subgroup difference differed from the P3a and P3b findings. This neurophysiological evidence is consistent with the use of ERP parameters as objective indicators of cognitive dysfunction in adolescent MDD.

**TABLE 3:** Analysis of event‐related potential results in case group and control group.

	Mild‐to‐moderate depression group	Major depression group	Healthy control group	*F* value	*p* value
Latent period (ms)					
N1	99.50 ± 18.45	94.11 ± 18.61	95.05 ± 9.75	1.300	0.276
P2	162.02 ± 18.77	160.30 ± 19.09	154.19 ± 12.49	2.036	0.135
N2	217.19 ± 22.40	212.28 ± 26.74	193.45 ± 14.11	12.107	0.000**
P3a	293.74 ± 25.55	295.67 ± 23.02	277.91 ± 9.35	6.353	0.002**
P3b	348.35 ± 42.28	357.04 ± 35.63	324.76 ± 6.55	6.841	0.002**
Amplitude (uV)					
P3a	8.98 ± 6.36	6.08 ± 4.62	8.80 ± 1.12	3.320	0.040*
P3b	6.74 ± 4.15	4.67 ± 3.41	7.39 ± 1.31	4.995	0.008**

^*^Data are presented as mean ± standard deviation. *p*‐values were calculated using ANOVA. *p < 0.05 and *p < 0.001 indicate significant differences between groups.

Abbreviation: ERP, event‐related potential.

### 3.4 Analysis of T Lymphocytes and Their Subsets and Inflammatory Cytokines

Serum levels of IL‐1β and IL‐6, as well as the percentages of CD3^+^CD4^+^ and CD3^+^CD8^+^ T lymphocyte subsets, were significantly higher in both mild‐to‐moderate and severe MDD groups compared to healthy controls (*p* < 0.05). In contrast, there were no significant group differences in TNF‐α concentration, CD45^+^ cells, CD3^+^ cell percentages, or the CD4^+^/CD8^+^ ratio (*p* > 0.05) (Table 4). These results suggest selective upregulation of pro‐inflammatory cytokines and T cell subtypes in adolescents with MDD.

**TABLE 4:** Analysis of T lymphocytes, their subsets, and inflammatory cytokines in case group and control group.

	Mild‐to‐moderate depression group	Major depression group	Healthy control group	*F* value	*p* value
TNF‐α (pg/mL)	12.89 ± 9.39	11.46 ± 7.42	6.33 ± 1.40	2.047	0.134
IL‐1β (pg/mL)	7.28 ± 2.93	8.45 ± 2.30	5.78 ± 1.88	7.933	0.001**
IL‐ 6 (pg/mL)	5.04 ± 1.45	6.34 ± 1.36	4.58 ± 1.70	11.037	0.000**
CD45+ cells(%)	36.22 ± 6.83	38.23 ± 5.56	31.84 ± 10.83	1.740	0.190
CD3+ cells (%)	74.30 ± 5.37	72.53 ± 6.05	76.26 ± 4.74	0.994	0.380
CD3+CD4+ cells (%)	40.17 ± 8.99	49.95 ± 8.65	33.65 ± 4.18	9.918	0.000**
CD3+CD8+ cells (%)	27.98 ± 7.68	36.12 ± 9.52	25.10 ± 10.56	4.933	0.013*
CD4+/CD8+	1.57 ± 0.65	1.47 ± 0.44	2.05 ± 0.88	1.901	0.164

^*^Data are presented as mean ± standard deviation. *p*‐values were calculated using ANOVA. *p < 0.05 and *p < 0.001 indicate significant differences between groups.

Abbreviations: IL‐1β, interleukin‐1 beta; IL‐6, interleukin‐6; TNF‐α, tumor necrosis factor‐alpha.

To improve interpretability, boxplots were generated to visualize the group‐wise distributions of TNF‐α, IL‐1β, and IL‐6 levels (Figure 1). IL‐1β and IL‐6 concentrations were markedly elevated in the major depression group relative to healthy controls, while TNF‐α levels did not differ significantly among groups.

**FIGURE 1:**
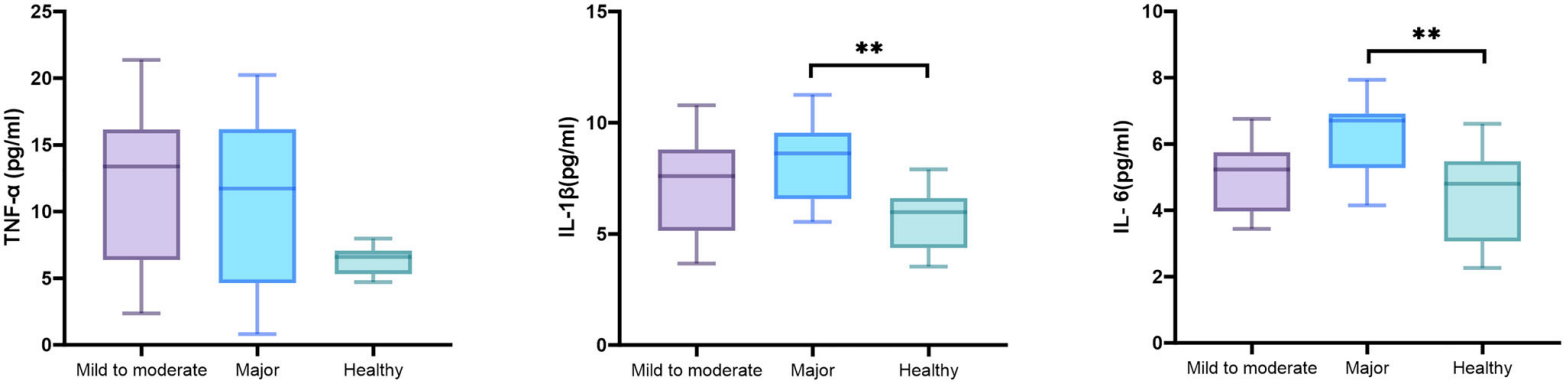
Boxplots of serum pro‐inflammatory cytokines (TNF‐α, IL‐1β, and IL‐6) across three groups: mild‐to‐moderate depression, major depression, and healthy controls. IL‐1β and IL‐6 levels were significantly higher in the major depression group compared to the healthy control group (*p* < 0.01).

Furthermore, Spearman correlation analysis within the MDD group showed that the percentage of CD3+ cells was positively correlated with MoCA scores (*r* = 0.388, *p* < 0.05), indicating that higher CD3+ cell percentages were associated with better cognitive function. Additionally, the percentage of CD3+CD8+ cells was positively correlated with HAMD17 scores (*r* = 0.422, *p* < 0.05), suggesting that higher CD3+CD8+ cell percentages were associated with more severe depressive symptoms. See Table 5 for details.

**TABLE 5:** Correlation between emotions, cognitive functions, and T lymphocytes.

		HAMD17	HAMA	MoCA
CD45+cells (%)	Correlation coefficient	0.246	0.226	−0.163
	*p* value	0.161	0.198	0.356
CD3+cells (%)	Correlation coefficient	−0.206	−0.270	0.388*
	*p* value	0.243	0.123	0.023
CD3+CD4+ cells (%)	Correlation coefficient	0.322	0.100	−0.069
	*p* value	0.063	0.575	0.700
CD3+CD8+ cells (%)	Correlation coefficient	0.422*	0.264	−0.260
	*p* value	0.013	0.131	0.138
CD4+/CD8+	Correlation coefficient	−0.244	−0.214	0.221
	*p* value	0.164	0.224	0.209

^*^Correlation coefficients and *p*‐values were calculated using Spearman's correlation analysis. *p* < 0.05 indicates a significant correlation. Abbreviations: HAMA, Hamilton Anxiety Scale; HAMD17, 17‐item Hamilton Depression Scale; MoCA, Montreal Cognitive Assessment Scale.

In addition to T lymphocyte markers, we conducted exploratory correlation analyses between pro‐inflammatory cytokines and clinical measures. As shown in Table 6, IL‐1β exhibited a trend toward positive correlations with both HAMD17 (*r* = 0.241, *p* = 0.058) and HAMA scores (*r* = 0.192, *p* = 0.101), and a weak negative correlation with MoCA scores (*r* = −0.187, *p* = 0.109), though none of these reached statistical significance. Similarly, IL‐6 showed a trend‐level inverse correlation with cognitive performance (MoCA: *r* = −0.218, *p* = 0.069), and weak positive associations with HAMD17 and HAMA scores (*r* = 0.163 and 0.155, respectively; both *p* > 0.05). While these correlations were not statistically significant, they are consistent with a possible association between systemic inflammation and both depressive symptomatology and cognitive function, consistent with prior neuroimmune research. These findings warrant further investigation in larger, adequately powered samples.

**TABLE 6:** Correlation between emotions, cognitive functions, and inflammatory cytokines.

		HAMD17	HAMA	MoCA
IL‐1β	Correlation coefficient	0.241	0.192	−0.187
	*p* value	0.058	0.101	0.109
IL‐6	Correlation coefficient	0.163	0.155	−0.218
	*p* value	0.145	0.167	0.069

^*^Correlation coefficients and *p*‐values were calculated using Spearman's correlation analysis. *p* < 0.05 indicates a significant correlation. Abbreviations: HAMA, Hamilton Anxiety Scale; HAMD17, 17‐item Hamilton Depression Scale; MoCA, Montreal Cognitive Assessment Scale.

These findings suggest that immune markers may show heterogeneous associations with cognitive performance and symptom severity across individuals. This pattern highlights the complex immune‐cognitive associations in adolescent MDD, aligning with our aim to explore multi‐level biomarkers.

However, these findings should be interpreted with caution. The present results demonstrate associations among depressive symptoms, cognitive performance, and immune‐inflammatory indicators, but they do not determine the direction of these relationships. In particular, the current data cannot establish whether immune‐inflammatory activation contributes to cognitive impairment, whether cognitive dysfunction aggravates depressive symptoms and subsequently affects immune status, or whether these changes occur simultaneously as part of a shared neurobiological process.

## 4 Discussion

Our study found that adolescents with MDD exhibited significant cognitive impairments, particularly in attention, delayed recall, and executive function, as evidenced by lower MoCA scores compared to healthy controls. This finding is consistent with previous research indicating that cognitive deficits are common in adolescents with depression and may persist even after depressive symptoms improve (Wu et al. 2024). Cognitive impairment in this population is concerning, as it can negatively impact academic performance, social functioning, and overall quality of life. Early identification and intervention are crucial to mitigate these effects.

ERP analysis revealed prolonged latencies and decreased amplitudes in the MDD group, particularly for N2, P3a, and P3b components (Sun et al. 2022; Wakode et al. 2022). These findings suggest delays in cognitive processing and reduced neural resources allocated to attentional and cognitive tasks. Previous studies have similarly shown that ERP components such as P300 may serve as candidate indicators for cognitive impairment in depression (Hanna et al. 2022; Santopetro et al. 2025; Arıkan et al. 2024; Kim et al. 2021). The prolonged latencies observed in our study may reflect slower processing speed, while decreased amplitudes could indicate reduced cognitive reserve or attentional resources. These ERP changes support the use of ERPs as candidate objective indicators of cognitive dysfunction in adolescent depression (Arıkan et al. 2024; Baran et al. 2023; Jänne et al. 2022).

We observed elevated levels of inflammatory cytokines (IL‐1β and IL‐6) and increased percentages of CD3+CD4+ and CD3+CD8+ T lymphocyte subsets in adolescents with MDD. These findings are consistent with prior evidence linking immune dysregulation and inflammation to MDD (Zhang et al. 2023; Chen et al. 2024; Peters et al. 2021; Ho et al. 2022). Notably, the percentage of CD3^+^ T cells was positively correlated with MoCA scores, suggesting that a higher proportion of total T lymphocytes was associated with better cognitive performance. While this finding may appear counterintuitive—given that elevated immune activation is often linked to cognitive dysfunction—it may reflect a compensatory immune surveillance mechanism rather than a pathological inflammatory state. Some studies have proposed that preserved or regulated peripheral T cell responses, particularly in youth, may be associated with maintaining neuroimmune homeostasis (Smolders et al. 2022), supporting neuroplasticity and synaptic integrity under chronic stress conditions. It is also possible that individuals with more robust immunological tone, including higher circulating CD3^+^ T cells, exhibit greater resilience against cytokine‐induced neurotoxicity, which could be associated with relative cognitive resilience.

This hypothesis aligns with emerging models of neuroimmune heterogeneity in depression, which posit that subgroups of patients may exhibit distinct immune‐cognitive profiles—ranging from inflammatory vulnerability to immunocompetent compensation. Longitudinal studies combining peripheral immune markers with functional imaging or CSF cytokine data may help clarify the directionality and clinical relevance of this relationship in future work (Lynall et al. 2020; Ambree et al. 2019).

The higher prevalence of depression in females compared to males during adolescence is well established (Hagatulah et al. 2024). This gender disparity may be attributed to a combination of biological, psychological, and social factors. For instance, hormonal changes during puberty and differences in brain development could contribute to the increased vulnerability of females to depression (Hurtado et al. 2024). Additionally, females may experience higher levels of stress and social adversity, which can exacerbate depressive symptoms (Crawford et al. 2024). Despite these differences, our study found no significant gender differences in cognitive function or immune inflammation within the MDD group. This suggests that the observed cognitive impairments and immune changes may be more related to depression itself rather than gender differences.

Importantly, although inflammatory cytokines and ERP changes show promise as potential biomarkers, our study did not perform receiver operating characteristic (ROC) analysis or external validation. Therefore, we do not claim their diagnostic utility at this stage. Further studies with larger sample sizes, external cohorts, and sensitivity/specificity analyses are necessary to evaluate their clinical applicability. Additionally, we did not conduct a priori sample size calculation due to limited effect size data in adolescent populations; however, post hoc power analysis based on our primary outcomes indicates adequate statistical power (≥0.80). Another important limitation is the cross‐sectional design of this study, which restricts causal inference. Although we observed significant associations among depression severity, ERP abnormalities, immune‐inflammatory alterations, and cognitive impairment, these findings cannot determine the temporal or causal direction among these variables. It remains unclear whether immune inflammation is prospectively associated with cognitive impairment, whether cognitive dysfunction is associated with worsening depressive symptoms and thereby influences immune activity, or whether both are driven by shared neurobiological processes in adolescent MDD. In response to this limitation, we have revised the wording throughout the manuscript to avoid causal statements and to emphasize association rather than causation. Future longitudinal studies should incorporate mediation and moderation analyses to further clarify the potential relationship of “depression severity–immune inflammation–cognitive impairment.” For example, inflammatory cytokines or T lymphocyte subsets may be tested as mediators between depression severity and cognitive decline, while ERP parameters may be examined as neurophysiological indicators linking immune dysregulation and cognitive dysfunction. Such analyses would help determine whether immune‐inflammatory abnormalities play a mechanistic, moderating, or merely associated role in cognitive impairment among adolescents with MDD.

In summary, our findings indicate that cognitive impairments in adolescents with MDD are associated with both electrophysiological changes and immune‐inflammatory activation. These results highlight the importance of integrating behavioral, neural, and immunological perspectives in understanding adolescent depression. Nevertheless, the present findings should be regarded as preliminary and associative. Further prospective studies are required to clarify the temporal and causal relationships among depression severity, immune inflammation, ERP abnormalities, and cognitive impairment. Future studies may evaluate whether immune‐modulating interventions or ERP‐based neurofeedback have clinical relevance for this population.

## Author Contributions

**Lu Yang**: Writing – review and editing, Writing – original draft, project administration, resources, data curation, methodology. **Zhenglai Liu**: data curation, investigation, validation, formal analysis. **Yunxin Ji**: supervision, software, conceptualization, validation. **Yiqin Huang**: investigation, validation, formal analysis, supervision. **Zhongze Lou**: software, data curation, investigation, validation. **Liemin Ruan**: conceptualization, software, data curation, investigation. **Li Zhang**: validation, formal analysis, supervision, visualization. All authors reviewed the results and approved the final version of the manuscript. All authors were informed each step of manuscript processing including submission, revision, revision reminder, and so on.

## Conflicts of Interest

The authors declare no conflicts of interest.

## Funding

The work was funded by the Zhejiang Provincial Medical and Health Science and Technology Plan Project (2023KY1073).

## Ethics Statement

This study was reviewed and approved by the Ethics Committee of the First Affiliated Hospital of Ningbo University (Ethics Approval No. 2022 Research No. 047A). All subjects and guardians fully understood and supported this study, and signed informed consent forms.

## Data Availability

The datasets used and analyzed during the current study are available from the corresponding author on reasonable request.
